# Supplementary material for: Comparison of genomic diversity between single and pooled Staphylococcus aureus colonies isolated from human colonization cultures
Source: Microb Genom. 2023 Nov 7;9(11):001111. doi: 10.1099/mgen.0.001111 (PMC10711313; doi:10.1099/mgen.0.001111)
Supplement: Supplementary material 1 [file mgen-9-1111-s001.pdf]

Fig S1

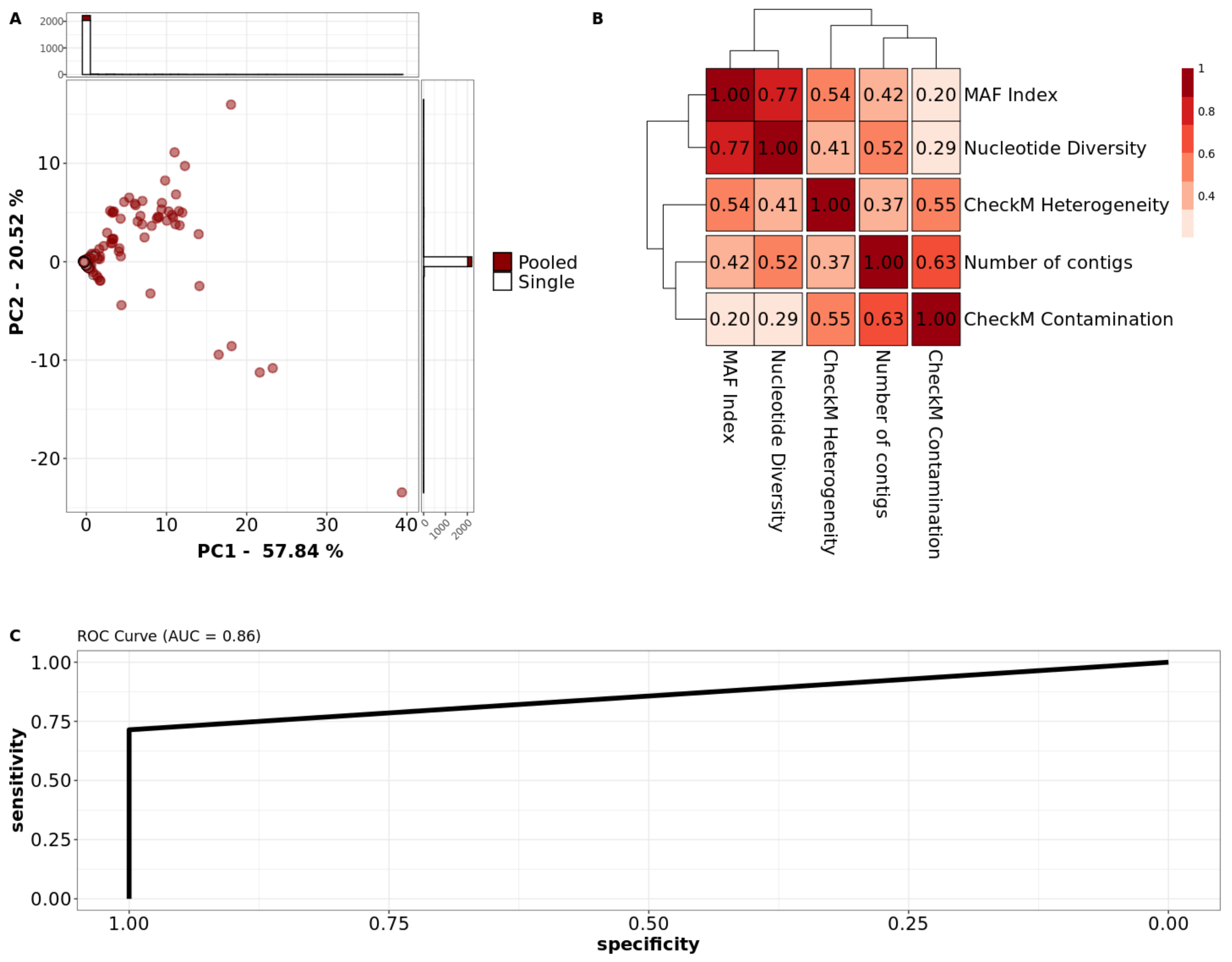

Fig S1 legend: Variation in pools was primarily driven by contamination and allelic diversity.

(**A**) PCA loading plot for principal components (PC) 1 (x-axis) and 2 (y-axis) explaining 70% of the total variance. White dots represent singles and red dots represent pools. 256 pools and 2032 singles were used for PCA. The density of the dots at their corresponding x and y positions are indicated by the histogram above and to the right of the plot respectively. The variance explained by each PC is indicated in the corresponding axis labels.

(**B**) Pearson's correlation coefficient matrix across five different diversity metrics. Each square indicates the Pearson *r* for comparing the corresponding parameters as labelled in the x and y axis. Scale indicates Pearson's *r* (Darker = higher *r*)

(**C**) Receiver operating characteristic (ROC) curve of the logistic model predicting multi-ST pools from parameters in **A** and **B**. Area under the curve (AUC) = 0.86.

Table S1

	PC1	PC2	PC3	PC4	PC5
**MAF Index**	0.462809	-0.5268234	0.1029145	-0.186782	-0.6802838
**Number of contigs**	0.4541359	0.288277	-0.5902644	-0.5812948	0.1560169
**CheckM Contamination**	0.4020166	0.6543029	0.0179086	0.5201767	-0.3733174
**CheckM Heterogeneity**	0.4418238	0.1308287	0.7578247	-0.2563222	0.3842866
**Nucleotide Diversity**	0.4719563	-0.4405963	-0.2576384	0.5393737	0.4752164

Table S1 legend: Summary of all five principal components (PC1 - PC5) for five parameters used in Fig S1. All 254 pools and 2032 singles were used for principal component analysis.

Fig S2

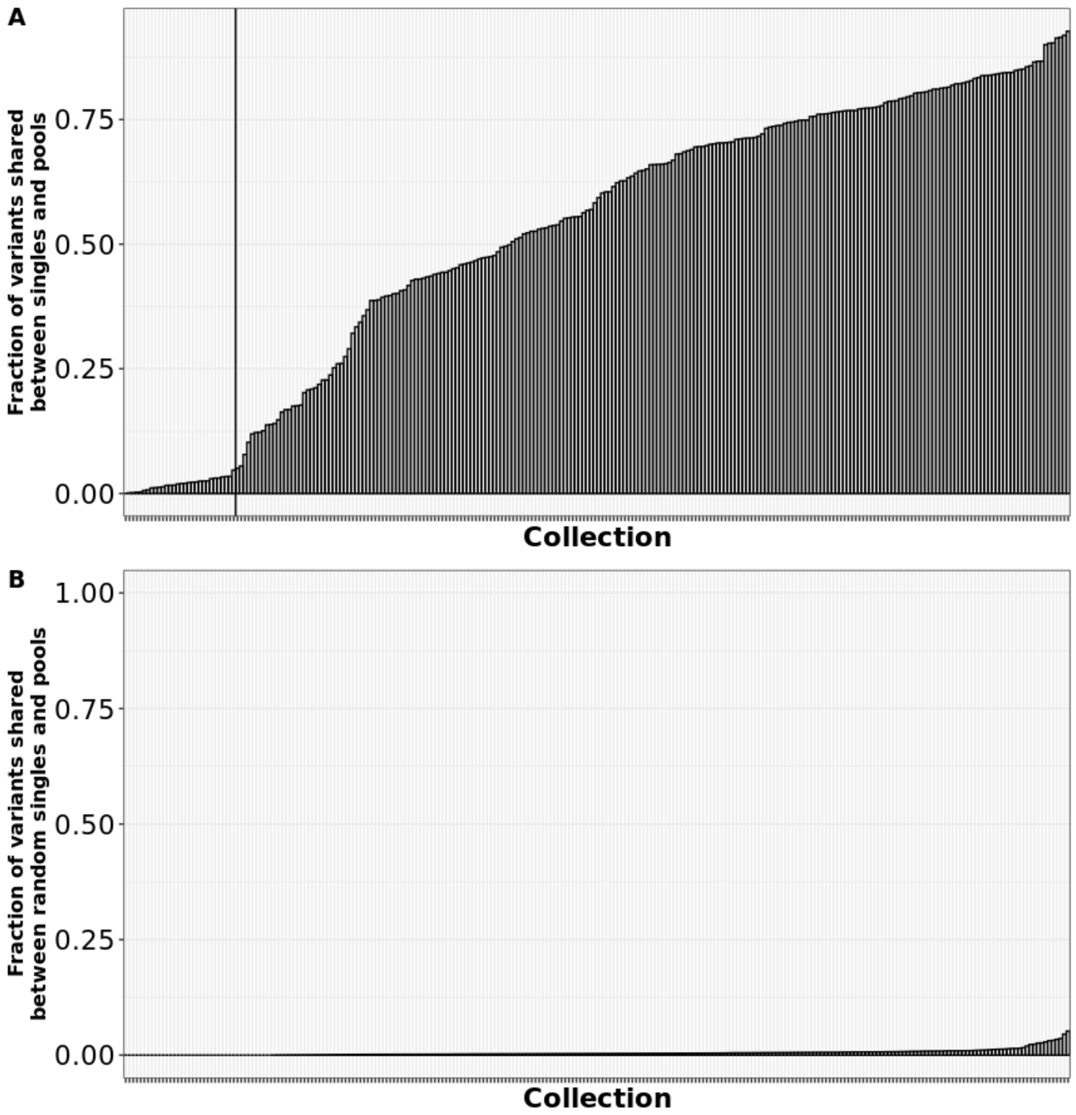

Figure S2 legend: Collections with <5% of their total variants shared between pools and singles were discarded.

(**A**) **Number of shared allelic sites revealed differences in the amount of diversity captured by single colonies and pools.** Each bar indicates a collection, and the height of the bar indicates the fraction of variants shared between the pools and at least one of the eight corresponding singles. Black vertical line indicates the threshold for shared fraction below which the singles and pools are not from the same sample (< 5% of variants shared)

(**B**) **Expected fraction of allelic sites shared between a pool and a random collection of eight singles**. Each bar indicates a collection and the height of the bar indicates the fraction of variants shared between the pool and at least one of eight singles from a random other collection. The maximum observed fraction did not exceed ~5% after 10 repetitions.

Fig S3

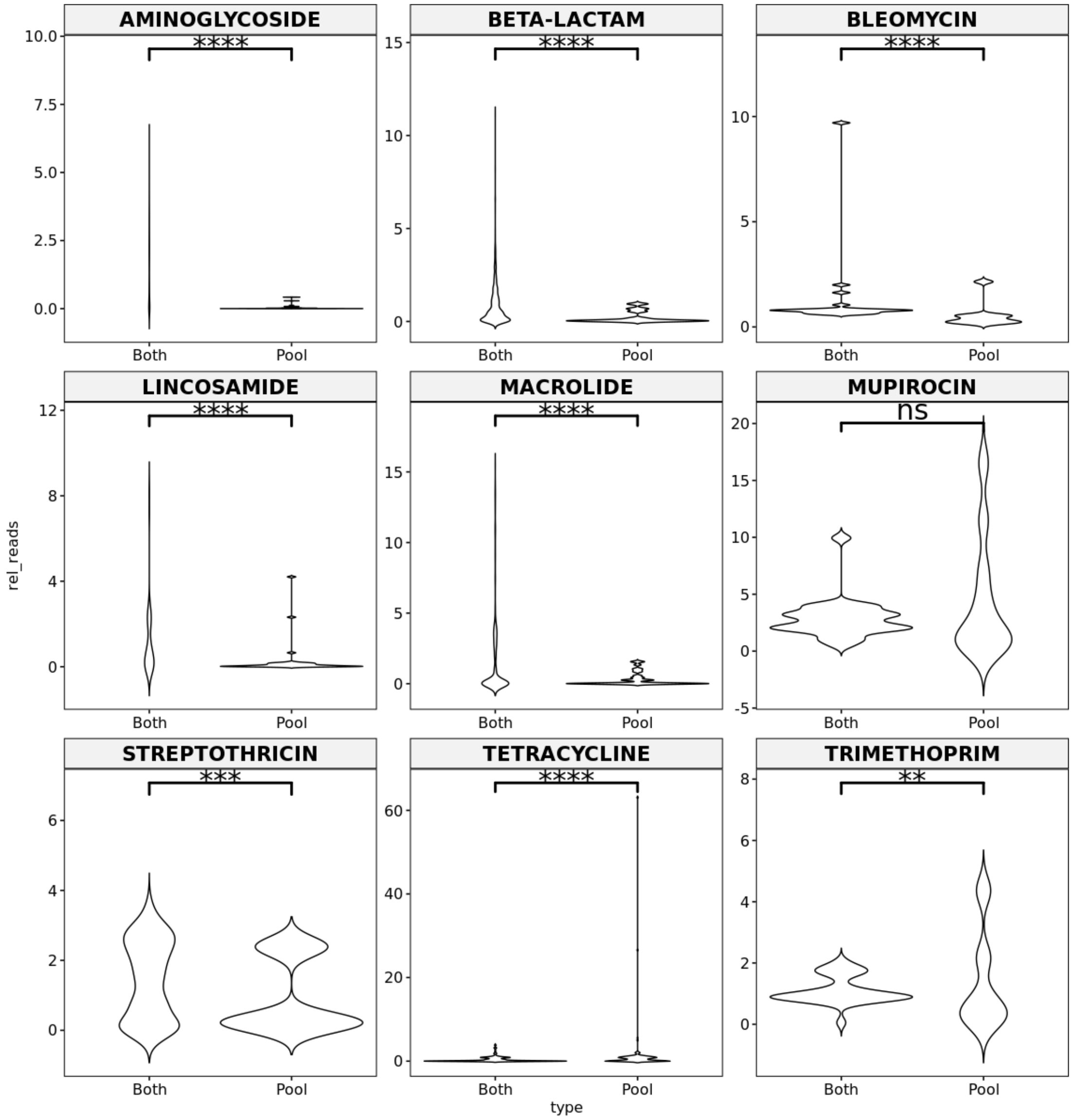

Fig S3 Legend: Mean read abundance is lower for AMR genes present only in the pools compared to AMR genes present in both pools and singles.

For each class of AMR, we estimated the number of reads mapped to each AMR gene in the AMRFinder database relative to the number of reads mapped to *rpoD* (relative copy number). All genes were normalised to 1 kb. For each AMR class, the relative copy number of genes found in both the pool and the corresponding single (“Both”) were compared against genes for the same AMR class found only in the pool (“Pool”) using Wilcoxon rank sum test with Bonferroni correction. ns = $p > 0.01$; ** = $p < 0.001$; *** = $p < 0.0001$; **** = $p < 0.00001$.

Fig S4

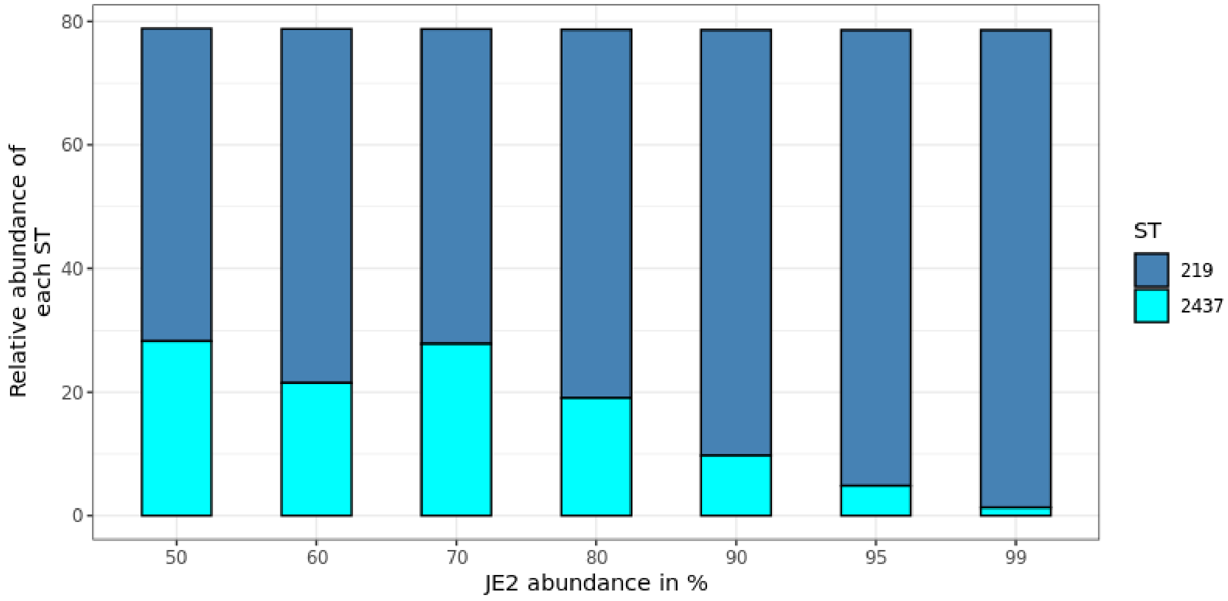

Fig S4 Legend: StrainGE (https://github.com/broadinstitute/StrainGE) reported a ST8 and ST5 mixture as ST219 and ST2437.

We generated artificial reads from a ST8 *S. aureus* reference (JE2, accession GCF_000013465.1) and a ST5 *S. aureus* reference (N315, accession GCF_000013425.1) reference genomes and mixed them in varying ratios using InSilicoSeq v1.5.4. The abundance of one of the strains, ranged from 50% of the total number of reads to 99%. Then, for each of these mixtures, we ran StrainGE v1.3.7 (1) using the default workflow described in the StrainGE documentation page. We built our reference database using 380 diverse *S. aureus* genomes from the Staphopia database each representing a unique ST, called non-redundant diversity (NRD) set (2), which includes ST5 and ST8. However, StrainGE mapped reads from our artificial ST8 and ST5 mixture to only ST219 and ST2437. While this is only a preliminary test run, this shows that performing strain level characterization requires significant additional testing and benchmarking with the appropriate controls which is beyond the scope of this study.

1. van Dijk LR, Walker BJ, Straub TJ, Worby CJ, Grote A, Schreiber HL, et al. StrainGE: a toolkit to track and characterize low-abundance strains in complex microbial communities. Genome Biol. 2022 Mar 7;23(1):74.

2. Iii RAP, Read TD. Staphylococcus aureus viewed from the perspective of 40,000+ genomes. PeerJ. 2018 Jul 12;6:e5261.
